# Simultaneous Effects of Viral Factors of Human Papilloma Virus and Epstein-Barr Virus on Progression of Breast and Thyroid Cancers: Application of Structural Equation Modeling

**DOI:** 10.31557/APJCP.2020.21.5.1431

**Published:** 2020-05

**Authors:** Shayan Mostafaei, Anoshirvan Kazemnejad, Amir Hossein Norooznezhad, Behzad Mahaki, Mohsen Moghoofei

**Affiliations:** 1 *Medical Biology Research Center, Health Technology Institute, Kermanshah University of Medical Sciences, Kermanshah, Iran. *; 2 *Department of Biostatistics, Faculty of Medical Sciences, Tarbiat Modares University, Tehran, Iran. *; 3 *Regenerative Medicine Research Center, Kermanshah University of Medical Sciences, Kermanshah, Iran.*; 4 *Department of Biostatistics, School of Health, Kermanshah University of Medical Sciences, Kermanshah, Iran. *; 5 *Department of Microbiology, Faculty of Medicine, Kermanshah University of Medical Sciences, Kermanshah, Iran.*

**Keywords:** Breast cancer, Thyroid cancer, Epstein-Barr Virus, Human Papilloma Virus, inflammation

## Abstract

This study aimed to assess effects of the sets of EBV and HPV expressed proteins simultaneously on the sets of cellular/inflammatory factors in breast and thyroid cancers using structural equation modeling. In this multi-center case-control study, according to the inclusion and exclusion criteria, 83 breast and 57 thyroid specimens were collected from the eligible patients. In addition, 31 and 18 histopathological evaluated normal breast and thyroid samples were also examined as age-matched healthy controls. In addition, ELISA and Real-time PCR were used to measure the expression level of viral and cellular/inflammatory genes and proteins. Structural equation modeling was used to test the causal associations between the sets of EBV and HPV expressed proteins with inflammatory factors in breast and thyroid cancers development. Breast cancer patients had a higher incidence of HPV-positively and EBV-positively than healthy controls (OR=1.66, 95%CI=0.79-3.47, P-value=0.177), (OR=3.18, 95%CI=1.52-6.63, P-value=0.002), respectively. In addition, thyroid cancer patients had a significantly higher incidence of EBV-positivity than healthy controls (OR=3.72, 95% CI=1.65-8.36, P-value=0.001). After fitting the SEM model, HPV proteins factor has significant direct and total effects on the cellular/inflammatory factors in breast cancer (direct effect: β=0.426, P-value=0.01; total effect: β=0.549, P-value<0.001). However, EBV proteins factor has most significant total effect on the cellular/inflammatory factors in breast cancer (total effect: β=0.804, P-value<0.001) than the cellular/inflammatory factors in thyroid cancer (total effect: β=0.789, P-value<0.001). For the first time, a significant association between *EBV* and *HPV* -genes, anoikis resistance and the development of breast and thyroid cancers demonstrated by using SEM, Simultaneously.

## Introduction

Breast cancer is the most common cancer among women which knowns as the third leading cause of death in this population. According to the data, an increasing incidence for this malignancy is expected in the next years (Anastasiadi et al., 2017; Siegel et al., 2019). Thyroid cancer has been described as the most common primary endocrine malignancy in woman globally (Ferlay et al., 2010) which has increased significantly in recent years (Stamatiou et al., 2016). As well as many other diseases, different etiologies other than genetic predisposition are involved in cancers. One of these important risk factors are infections, especially those caused by viral agents (Parkin, 2006; Etemadi et al., 2017). Previous studies have shown the association between some viruses such as human papillomavirus (HPV) and Epstein-Barr virus (EBV) with thyroid and breast cancer respectively by presence of their genome and proteins in the obtained tumoral tissues from patients (OTTP) (Kan et al., 2005; Khodabandehlou et al., 2019). Furthermore, it has been proved that viruses could involve in tumor development through different pathways such as chronic inflammation, disruption in cellular regulatory factors, and resistance to natural cell death (Mesri et al., 2014).

The HPV genome has been composed of 3 segments including early region (E), and late region (or L which consists of L1 and L1), and long control region (LCR) (Morshed et al., 2014). The E region encodes E1, E2, E4, E5, E6, and E7 which among them E6 and E7 have been shown as the oncoproteins that playing role in resistance to apoptosis and promotion of the cell proliferation (Jackson et al., 2000; Zur Hausen, 2002). As well as HPV, some of EBV genes such as Epstein–Barr nuclear antigen 1 (EBNA1), EBNA2, Epstein-Barr Virus-Encoded RNA 1 (EBER1), EBER2, BamHI A rightward transcript (BARTs), Latent Membrane Protein 1 (LMP-1), and LMP-2 have been proved to be carcinogenesis and being involved in tumor development (Pang et al., 2009).

Among HPV proteins, E6 and E7 are very important as carcinogenic and tumor development agents. E6 protein interacts with two important cellular regulatory factors named p53 and BAK34. These interactions have two very important consequences, leads to cellular resistance to apoptosis and increase of chromosomal instability (Jackson et al., 2000). Also, E7 could interact with retinoblastoma (RB) and through the degradation of this protein. RB binds to the E2F1 which is a transcription factor and therefore, prevent it to act in the cellular transcription machinery (Zur Hausen, 2002; Yim and Park, 2005). Moreover, HPV proteins, especially E6 and E7, are able to interact with breast and ovarian cancer susceptibility gene-1 (BRCA1) and BRCA2 that are tumor suppressors agents (Zhang et al., 2005).

The role of viral proteins in chronic inflammation has been confirmed (Mesri et al., 2014; Etemadi et al., 2017; Khodabandehlou et al., 2019). Viral persistent infections could lead to chronic inflammation which is characterized by secretion and/or expression of inflammatory cytokines as well as reactive oxygen and nitrogen species (RONS). This induced chronic inflammation could suppress anti-tumoral immunity and initiate and promote tumoral progression as well as metastasis (Stone et al., 2014; Esquivel-Velázquez et al., 2015; Fernandes et al., 2015). Chronic inflammation enhances tumor development through different routes such as secretion of the growth factors, angiogenesis, and tissue remodeling (Goldszmid et al., 2014). Different cytokines such as transforming growth factors like beta (TGF-β), Interleukins (ILs), and tumor necrosis factor α (TNF-α) could stimulate breast and thyroid cancers cellular proliferation and/or invasion (Nicolini et al., 2006; Mesri et al., 2014; Esquivel-Velázquez et al., 2015; Etemadi et al., 2017; Khodabandehlou et al., 2019). Other involved factors in inflammation are NF-κB and RONS that have a pivotal role in initiating and development of solid tumors (Haklar et al., 2001; Lu et al., 2014; Etemadi et al., 2017).

Based on the results of the previous studies, there are possible associations between breast and thyroid cancers (Giani et al., 1996; Smyth, 1997; Chen et al., 2001; Moghoofei et al., 2019) as well as the between EVB and HPV proteins (Wong et al., 2002; Al Moustafa et al., 2009). Furthermore, the effects of the viruses and their expressed proteins on the cancers have been demonstrated. Therefore, assessing simultaneous, group, and casual effects of HPV and EBV viral factors on the cellular signaling and inflammatory factors based on the conceptual diagram of associations by structural equation modeling (SEM) could be very helpful to investigate the involved molecular pathways of viral infections in cancers. SEM as a multivariate multiple regression allows one to estimate the strength and sign of direction and indirection associations for complicated causal schemes with multiple dependent and independent variables. The SEM has several advantages such as detection of most important viral and cellular factors in occurrence and progression of the cancers and it allows to calculate direct, indirect, and total effects (Sobel, 1987). In this study, the possible role of HPV and EBV in breast cancer and EBV in thyroid cancer were assessed.

## Materials and Methods

*Study Design and Study Subjects*

This multi-center case-control study was performed between January 2015 and April 2017 in two hospitals of different cities in Iran (Kashani Hospital, Shahrekord and Rasoul-e Akram Hospital, Tehran). The current study was approved by Medical Ethics Committee of affiliated University) Authors adhered to the 1975 Helsinki declaration and its next revisions. Also, all the human subjects freely signed a printed consent form after explanation of the study’s aim and methods according to their level of knowledge. The inclusion criteria were defined as patients with approved histopathological (core/fine needle biopsy) evidence of breast/thyroid cancer and accessibility of fresh tissue samples. No limitations in age, type of cancer, tumor size, and stage were considered for the patients. In addition to, different parameters such as past or current medical history of chemotherapy and/or radiotherapy, being pregnant, biologic anti-cancer therapies, and systemic inflammatory diseases such as rheumatoid arthritis were defined as exclusion criteria. Furthermore, 31 normal breast and 18 normal thyroid tissues with normal histopathological evaluations were also examined as age-matched healthy controls. All the controls were healthy with no history of estrogen therapies, oral contraceptive consumption, cervical cancer, and smoking. According to the inclusion and exclusion criteria, 83 breast and 57 thyroid specimens were collected from the patients in the mentioned period. All tissue samples were snap-frozen and fresh tissue which were stored at -80°C. Furthermore, the stage of the tumors according to the TNM system was provided by consulting an expert cancer team consists of an oncologist, a radiologist, and a cancer surgeon.

*Nucleic Acid Extraction*

 DNA and total RNA extraction were performed by QIAamp Fast DNA Tissue Kit (Cat# 51404, QIAGEN, Germany) and RNeasy Mini Kit (Cat# 74104, QIAGEN, Germany) respectively according to the manufacturers’ instructions.

*EBV and HPV Detection and Genotyping*

In order to evaluate the EBV infection in the OTTP, nested-polymerase chain reaction (PCR) was used with the same set of primer previously described by Shimakage et al. (Shimakage et al., 2003). Reactions were including 2x Taq Master Mix (Cat# M0270L, New England BioLabs, Massachusetts, USA), 10 nM of each primer (Metabion, Germany), 1 µg of template DNA and water up to 25 μl. Amplification of samples was done by 45 cycles of PCR with the temperatures explained by Shimakage et al (Shimakage et al., 2003). Moreover, for EBV typing EBNA2 primers were used (van Baarle et al., 1999).

Detection of HPV infection in the OTTP was performed by PCR method using primers for L1 and E7 genes as previously reported by Kroupis et al., (2006). HPV typing was performed based on the method described by Khodabandehlou et al., (2019).

*Expression level of Cellular and Viral Factors*

*E2*

*E2* expression level was investigated by QuantiFast SYBR^®^ Green PCR Kit (Cat# 204054, QIAGEN, Germany) kit. The used primers for the amplification of *E2* were (Webster et al., 2000): 

Forward primer:5’-CTACGAATTCATGGAGACTCTTTGCCAACG-3′. Forward primer: 5’-GATAGAATTCTCATATAGACATAAATCCAG-3′ 

*E6*

In order to evaluate E6 gene expression level, one-step RT-PCR® kits (Cat#210212, QIAGEN, Hilden, Germany) were used with following primers (Jang et al., 2011): 

Forward 5’-GCAATGTTTCAGGACCCACA-3’ 

Reverse 5’-ACAGCATATGGATTCCCATCTC-3’

*E7*

*E7* gene expression assessment was performed by QuantiNova Reverse Transcription® Kit (Cat# 205410, QIAGEN, Hilden, Germany). The used primers and probe in this section were (Wang-Johanning et al., 2002): Forward primer: 5’-AAGTGTGACTCTACGCTTCGGTT-3’ everse primer: 5’-GCCCATTAACAGGTCTTCCAAA-3’ Probe: FAM-TGCGTACAAAGCACACACGTAGACATTCGTA-BHQ

*P53 and RB*

The levels of *p53* and *RB* were measured by Abcam’s p53 Simple Step ELISA® Kit (Cat# ab171571, Abcam, Cambridge, MA, USA) and Human Retinoblastoma ELISA® kit (Cat# CS0050, Sigma-Aldrich, Saint Louis, USA) respectively, according to the manufacturer’s instructions.

*BRCA1 and BRCA2*

The levels of BRCA1 and BRCA2 were measured by BRCA1 and BRCA2 ELISA Kits (Human) (Cat# MBS008497 and MBS2501553 respectively, My BioSource, Inc. CA, USA) according to the manufacture’s protocol.

*LMP-1 and LMP-2A gene expression*

The expression level of *LMP-1* gene in OTTP (thyroid) was measured by quantitative RT-PCR (qRT-PCR) (Cat# R-4000, qPCR Master Mix, GeNet Bio, Chungnam, Korea) using following primers and probes (Kubota et al., 2008): Forward primer: 5’-CCCTTTGTATACTCCTACTGATGATCAC-3’Reverse primer: 5’-ACCCGAAGATGAACAGCACAAT-3’ Probe: FAM-CTCATCGCTCTCTGGAATTTGCACGG-BHQ1a

Also, *LMP-2A* expression status was measured by SYBR Green PCR Master Mix Kit (Cat# 4309155, Applied Biosystems, Foster City, CA) as it has been described by Bell et al., (2006).

*EBERs gene expression*

For the investigation of *EBER-1* and *EBER-2 *expression levels in the OTTP (thyroid), qRT-PCR was used as Shannon-Lowe et al. have previously described (Shannon-Lowe et al., 2009).

*Cytokines assay*

For evaluation of IL-1, IL-6, IL-10, and IL-17 levels, enzyme-linked immunosorbent assay (ELISA) was used with human IL-1, IL-6, IL-10, and IL-17 ELISA^®^ Kits (Cat# respectively: ab100562, ab46027, ab46034, and ab83668, Abcam, Cambridge, MA, USA) respectively as the manufacture’s protocols have described. Also, levels of Bcl-2 and NF-kB were measured using ELISA method (Bcl-2 and NFkB p65 Transcription Factor Assay^®^ Kit (Cat# respectively: ab119506 and ab133112, Abcam, Cambridge, MA, USA)). Moreover, the level of TNF-α and TGF-β were measured by Human TNF Alpha PicoKine™ ELISA Kit (Cat# EK0525, Boster Biological Technology, Pleasanton CA, USA) and Human TGF-beta 1 Quantikine ELISA^®^ Kit (Cat# DB100B, Minneapolis, MN, USA), respectively, according to the manufacturers’ instructions.

*Reactive Oxygen Species and Reactive Nitrogen Species*

The Reactive Oxygen Species (ROS) and Reactive Nitrogen Species (RNS) levels were measured by OxiSelect™ Intracellular ROS/RNS Assay kit (Cat# STA-347-CB, Cell Biolabs, Inc., San Diego, CA) following the manufacture’s protocol.

*Survivin and CD44*

ELISA Kit for Survivin (Surv) (Cat# SEC045Hu, Houston, Texas, USA) was used for measurement of the survivin concentrations according to the manufacturer’s instructions. Should be noted that all measurements of this protein were performed in duplicate. Also, the expression levels of CD44 in OTTP (thyroid) were performed by qRT-PCR as Rajarajan et al. explained in their study (Rajarajan et al., 2012).

*Statistical Methods*

In the first step, Spearman’s rank correlation was applied, and stepwise linear regression was obtained to assess the associations between the studied variables and to implement the path diagram. Next step, SEM was applied to examine the path diagram. Structural equation modeling includes causal modeling, analysis of covariance structures, and latent variable models. This model is a generalization of multivariate multiple regression that allows one to estimate the strength and sign of direction and indirection association for complicated causal schemes with multiple dependent and independent variables (Tomarken and Waller, 2005). The SEM has several advantages like effect decomposition, it allows for calculating direct, indirect and total effects, and ability of SEM to show by path diagram and so on (Wolfle, 1980). According to the Kline’s suggestion and the measure of sampling adequacy for each variable in the model, 189 cases were included in this analysis, the sample size is adequate for this SEM (Kline, 2015). Standardized coefficients (β) as the effect size of associations were calculated. Goodness of fit (GOF) indices (e.g. The Root Mean Square Error of Approximation (RMSEA), the goodness of fit index (GFI), the adjusted GFI) were applied for assessing of the fitness of the model. Structural equation modeling (SEM) was fitted by maximum likelihood estimation (MLE) method. All of the statistical analysis was performed using “lavaan” R package and STATA 11.0 (STATA Corp, College Station, TX). In differential analysis, p-values were adjusted by Benjamini-Hochberg method for multiple testing. Any P-value less than 0.05 was considered as statistically significant. 

## Results

*Characteristics of Studied Subjects*

In this study, 83 female breast cancer subjects, including 10 (12.5%), 23 (27.8%), 37 (44.4%), 4 (4.2%), and 9 (11.1%) patients with medullary, invasive lobular, invasive and in-situ ductal, mucinous, and tubular carcinoma were examined, respectively. The mean age in the breast cancer group was 49.02±11.21 (ranged 30 to 81) years. From 57 patients diagnosed with thyroid cancer, 36.8% (21/ 57) were males with the mean age of 48.81±12.53 (ranged 27 to 81) years. In the patients diagnosed with thyroid cancer, histopathologic evaluations showed 41 (71.9%), 9 (15.8%), 4 (7%) and 3 (5.3%) papillary, follicular, undifferentiated, and medullary carcinomas respectively. Also, 31 healthy individuals with the mean age of 49.34±11.01 (ranged from 24 to 72) years were evaluated as breast cancer pair control group. It was shown that patients with breast cancer and their healthy controls were age-matched (*P*-value=0.934). From 18 healthy individuals in the thyroid control group, 38.9% (7/18) were males with the mean age 50.44 ±10.92 (ranged 24 to 64) years. Also, thyroid cancer and their healthy control group were age and sex-matched (*P*-value=0.62 and *P*-value=0.876, respectively). Patients with breast cancer had a non-significant higher incidence of positive HPV and a significant higher incidence of positive EBV results than their matched healthy controls (OR= 1.66, 95%C.I=0.79-3.47,* P*-value= 0.177 and OR= 3.18, 95% C.I=1.52-6.63, *P*-value= 0.002 respectively). In addition, patients with thyroid cancer had a significantly higher incidence of EBV infection than their matched healthy controls (OR= 3.72, 95%C.I=1.65-8.36,* P*-value= 0.001).

*Differential analysis of expression level of cellular signaling, inflammatory, and viral factors*

These results demonstrated that the expression levels of *LMP-1*, *LMP-2A*, *EBER-1*, and* EBER-2* were significantly higher in patients with breast cancer than their control samples (Fold changes>4, adj. *P*-values<0.001). Also, the expression levels of* EBER-1* and* EBER-2* were significantly higher in the thyroid cancer group compared to their controls (Fold changes=1.52, adj. *P*-value=0.001; Fold changes=1.39, adj. P-value=0.01, respectively). In addition to, the expression levels of *E6* and *E7* were significantly higher in patients with breast cancer in comparison to their control group (Fold changes=3.03, adj. *P*-value<0.001 and Fold changes=4.74, adj. P<0.001; Fold changes=3.38, adj. *P*-value=0.002 respectively). Other HPV evaluated genes did not show any significant changes in expression to the healthy controls (Table 1).

*Principal Component Analysis of cellular and viral factors for clustering*

**Figure 1:**
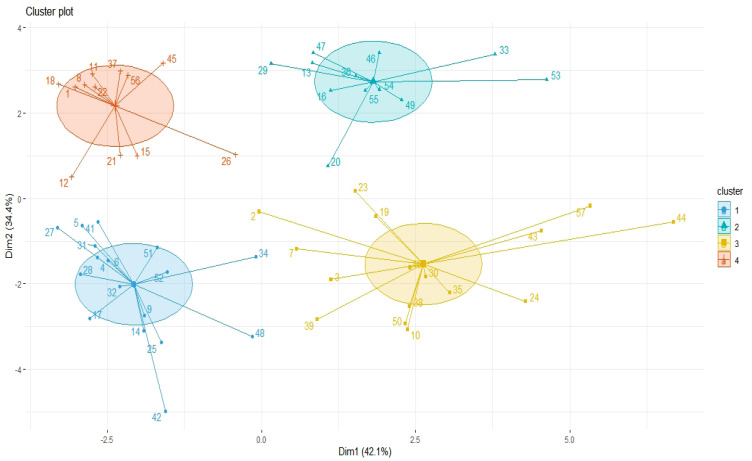
Principal Component Analysis (PCA) of Cellular, Inflammatory, and Epstein-Barr Virus (EBV) Factors for Clustering of 57 Thyroid Cancer Patients

**Figure 2:**
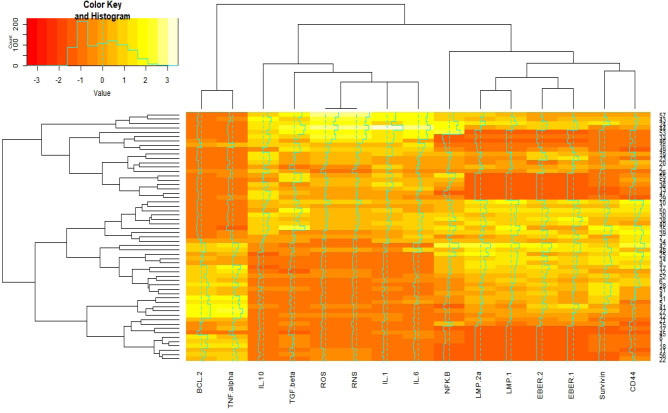
Interactive Cluster Heatmap Displaying Similarity in Distributions of Cellular Signaling, Inflammatory, and Epstein-Barr Virus (EBV) Factors (as Columns) and 57 Thyroid Cancer Subjects (as Rows), rows and columns of the heatmap have been reordered according to a hierarchical clustering, represented by the dendrogram

Results of Figure 1 indicate 57 thyroid cancer patients based on the co-expression patterns of cellular signaling and inflammatory factors were grouped by four clusters. EBV factors and surviving, CD44, and NFK-B by one-cluster and other cellular signaling and inflammatory factors by one major cluster have most similarity co-expression patterns (Figure 2).

**Figure 3:**
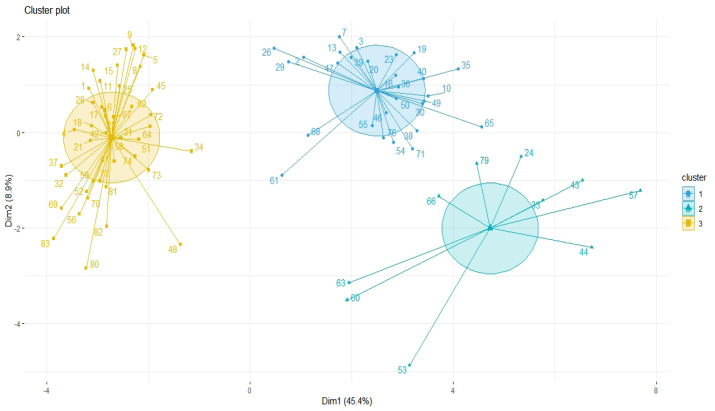
Principal Component Analysis (PCA) of Cellular/Inflammation, Human Papilloma Virus (HPV), EBV Factors for Clustering of 83 Breast Cancer Patients

**Figure 4:**
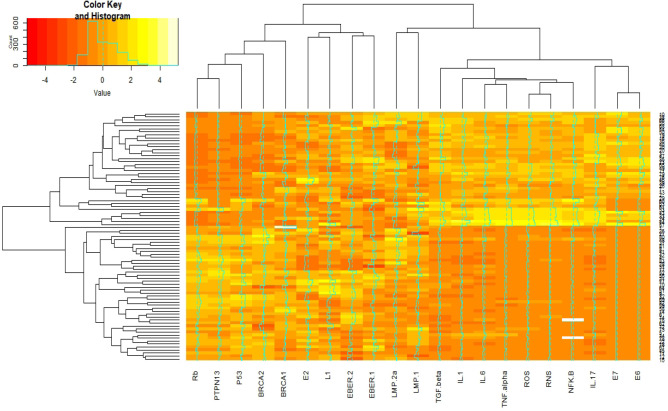
Interactive Cluster Heatmap Displaying Similarity in Distributions of Cellular Signaling, Inflammatory, Human Papilloma virus (HPV), and Epstein-Barr virus (EBV)factors (as columns) in 83 breast cancer subjects (as rows). Rows and columns of the heatmap have been reordered according to a hierarchical clustering, represented by the dendrogram

Results of Figure 3 indicate 83 breast cancer patients based on the co-expression patterns of cellular signaling and inflammatory factors were grouped by three clusters. E6, E7, IL-17, NFK-B, RNS, ROS, IL-1, IL-5, and TGF-beta by one-cluster, and other cellular signaling and inflammatory factors and EBV factors by another major cluster have most similarity co-expression patterns (Figure 4).

*Assessing the simultaneous, group, and casual effects of HPV and EBV viral factors on the cellular signaling and inflammatory factors in the development of breast and thyroid cancers by SEM*

**Figure 5:**
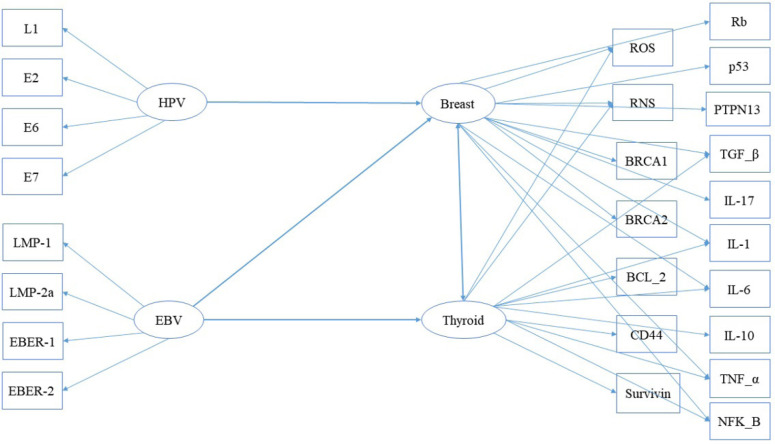
Path Diagram for Showing the Associations between Human Papilloma Virus (HPV), Epstein-Barr Virus (EBV), Cellular Signaling, and Inflammatory Factors in Breast and ThyroidCancers

Path diagram has been shown as Figure 5. After fitting the SEM model to studied data, HPV factors showed significant direct and total effects on the cellular signaling and inflammatory factors in the breast cancer group. Also, EBV factors showed the most significant total effect on the cellular signaling and inflammatory factors in breast than thyroid cancer (Table 2).

According to the path standardized coefficients (β) presented in Table 2, HPV factors have a non-significant indirect effect (β=0.123, P-value=0.09) on the cellular signaling and inflammatory factors in patients with breast cancer. However, these factors showed significantly direct and total effects on the cellular signaling and inflammatory factors in the breast cancer group (direct effect: β=0.426, P-value=0.01; total effect: β=0.549, *P*-value<0.001). 

On the other hand, EBV factors had direct (β=0.504, P-value<0.001), indirect (β=0.30, *P*-value=0.011), and total (β=0.804, P-value<0.001) significant effects on the cellular signaling and inflammatory factors in the breast cancer than thyroid group (direct effect: β=0.411, P-value<0.001; indirect effect: β=0.278, *P*-value=0.028; total effect: β=0.789, *P*-value<0.001) (Table 2). 

There are some of the fit indices to evaluate the model which all of them indicated that the model was acceptably fitted. The results of model fitness with an accepted range for evaluating the validity of the model have been reported in Table 3.

**Table 1:** Differential Analysis of Expression Level of the Viral Factors between Breast, Thyroid Cancer and Healthy Control Groups

Virus	Expressed gene	Breast Cancer (N=83)	Thyroid Cancer (N=57)	Healthy Breast (N=31)	Healthy Thyroid (N=18)	Fold Change*	Fold Change$	Adj. P-value*	Adj. P-value$
EBV†	*LMP-1*	12.36 ± 5.23	14.65 ± 6.78	2.39 ± 5.77	11.50 ±7.67	5.17	1.27	<0.001	0.1
	*LMP-2A*	10.55 ± 6.60	14.07 ± 6.19	2.48 ± 5.99	10.90 ± 7.58	4.25	1.29	<0.001	0.077
	*EBER 1*	14.02 ± 4.49	16.22 ± 6.05	2.74 ± 1.16	10.70 ± 6.89	5.11	1.52	<0.001	0.001
	*EBER 2*	12.60 ± 6.83	15.27 ± 5.73	2.68 ± 1.12	11.0 ± 6.76	4.7	1.39	<0.001	0.01
HPV‡	*L1*	0.29 ± 0.99	NA §	0.1 ± 0.25	NA	2.9	NA	0.19	NA
	*E2 *	0.79 ± 0.852	NA	0.48 ± 0.91	NA	1.62	NA	0.998	NA
	*E6*	4.90 ± 6.77	NA	1.03 ± 2.66	NA	4.74	NA	<0.001	NA
	*E7*	3.69 ± 5.18	NA	1.09 ± 3.07	NA	3.38	NA	0.002	NA

†, Epstein-Barr virus; ‡, Human papilloma virus; § NA, Not applicable; Geometric Mean± Standard Deviation; * Adj. p-values were corrected by Benjamini-Hochberg method for multiple comparisons. Bolded P-values indicated as statistical significant at level of 0.05. * Comparison between breast cancer versus healthy controls, $ Comparison between thyroid cancer versus healthy

**Table 2:** Direct, Indirect and Total Effects Obtained by SEM

	Cellular/inflammatory factors in Breast Cancer			Cellular/inflammatory factors in Thyroid Cancer		
Risk Factor	Direct	Indirect	Total	Direct	Indirect	Total
HPV† proteins	0.426*	0.123	0.549*	NA	NA	NA
EVB‡ proteins	0.504*	0.30*	0.804*	0.411*	0.278*	0.789*

†, Human papilloma virus; ‡, Epstein-Barr virus; Data presented as path standardized coefficients (β) for each cell; *, effect indicated as statistically significant at level of 0.05 (P-values <0.05); NA, Not applicable

**Table 3:** The Results of Evaluating the Goodness of Fit the Model Based on the Various Indices

Goodness of fitness	Model	Accepted Range
Normal Theory Weighted Least Squares Chi-Square	χ^2^/df =1.81, p-value=0.46	<2
Akaike information criterion (AIC)	191.407	-
Root Mean Square Error of Approximation	0.012	<0.08
Goodness of Fit Index (GFI)	0.989	>0.95
Adjusted Goodness of Fit Index (AGFI)	0.98	>0.90
Root mean square residual (RMR)	0.113	>0.08
Normed fit index (NFI)	0.987	>0.90
Relative fit index (RFI)	0.986	>0.95

## Discussion

In the current study, it was shown that the expression levels of *LMP-1*, *LMP-2A*, and *EBER-1*, *EBER-2 (EBERs) *were higher in breast cancer tissue compared to their matched healthy controls while in the same compartment was not significant for thyroid cancer (Table 1). The association between the expression of *LMP-*1,* LMP-2A, *and *EBERs* with survivin, *CD44*, *PTPN13*, and *Bcl-2 *have been reported previously (Port, 2013; Tsang and Tsao, 2015; Moghoofei et al., 2019). Having in mind that *EBERs* are involved in resistance to apoptosis (Li, 2015), our findings can indicate that the mentioned EBV gene products could exert different effects on the expression levels of anoikis inhibitory genes which consequently could lead to development of thyroid cancer. Moreover, as it was showed the mentioned EBV factors showed most direct (β=0.504, *P*-value<0.001), indirect (β=0.30, *P*-value=0.011), and total (β=0.804, *P*-value<0.001) significant effects on the cellular signaling and inflammatory factors in breast cancer than thyroid cancer group (direct effect: β=0.411, *P*-value<0.001; indirect effect: β=0.278, P-value=0.028; total effect: β=0.789, *P*-value<0.001) (Table 2). Interestingly, previously it has been shown that the expression levels of *EBV* genes (*LMP-1, LMP-2A, EBER-1, *and *EBER-2*) in thyroid tumor tissues were higher than healthy controls (Moghoofei et al., 2019).

Among the *HPV* genes, the expression levels of *E6 *and *E7* were higher in breast cancer tissues than controls while *E2* was not significantly different in the same groups (Table 1). The final products of this genes targets *p53* and *RB* (of the most important genes controlling cellular processes) and could disrupt the cell cycle, transformation initiation, and finally tumor development (Doorbar, 2006; Khodabandehlou, 2019). Previous studies demonstrated that *E6* and *E7* (as the antagonists) could interact with *BRCA1* and alter its activity. It has been shown that *BRCA1* and *BRCA2* are involved in the repair process of DNA damage in different tissues and any reduction and/or disruption of these two proteins may lead to cancer (Friedenson, 2007). Also, *BRCA1* is able to interact with the important regulatory cellular factors such as *RB* and *p53* (co-activator of p53-mediated transcription) which this interaction is necessary for G1 checkpoint in the cell cycle (Zhang, 2005). Therefore, HPV oncoproteins (E6 and E7) could exert their effects on the functions of *p53* and *RB* through *BRCA1* pathway. As Khadabandehlou et al. showed, the expression levels of *RB*, *p53*, *BRCA1*, and *BRCA2* were decreased in HPV-positive patients with breast cancer compared to same HPV-negative patients and healthy controls (p< 0.05) (Khodabandehlou, 2019). In the current study, according to the path standardized coefficients (β) presented in Table 2, HPV factors had a non-significant indirect effect (β=0.123, P-value=0.09) on the cellular signaling and inflammatory factors in patients with breast cancer. Although, these factors showed significantly direct and total effects on the cellular signaling and inflammatory factors in the breast cancer group (direct effect: β=0.426, P-value=0.01; total effect: β=0.549, P-value<0.001).

Another important aspect regarding the role of viruses in tumor development is chronic inflammation. This phenomenon affects different phases of tumor life including initiation, progression, and development (Goldszmid, 2014). Considering the role of inflammation in cancers, the TNF-α--NF-κB axis has been shown to induce the malignant behavior and invasiveness of breast cancer. IL-1, IL-6, TNF- α, and TGF-β, are the inflammatory cytokines with the potential of inducing different pro-angiogenic factors such as vascular endothelial growth factor (VEGF) which is the key element of angiogenesis (Esquivel-Velázquez, 2015). The role of viruses in inducing of inflammation and correlation of the induced inflammation with tumor development have been demonstrated in different studies (Coussens, 2002; Fernandes, 2015; Mesri, 2014). In this study, there was an association between increases in the expression levels of some *EBV* genes (*LMP-1* and *LMP-2A*) and increased level of expression in some inflammatory cytokines in patients with breast and thyroid cancers. Furthermore, Morris et al. indicated that *LMP-1* was associated with increased expression of the *IL-1* that involves in inflammation and several changes in host-cell gene expression (Morris et al., 2008). Furthermore, *LMP-1* is involved in the induction of several important regulatory factors such as NF-kB, inflammatory cytokines/chemokines, and growth factors (Etemadi et al., 2017). The inflammatory cytokines such as IL-1, IL-6, TNF- α, and TGF-β could induce cancer cells proliferation and tumoral invasion through activation of NF-κB (Khodabandehlou, 2019). Moreover, this study showed the association between expression levels of EBER-1 and EBER-2 with the evaluated inflammatory cytokines. In agreement with the current results, Li et al., (2015) demonstrated that inflammatory cytokines can be induced by EBERs gene products in nasopharyngeal carcinoma through TLR3 (Toll-like receptor 3) signaling pathway. In addition, their results approved that LMP-1 could lead to up-regulation of some inflammatory cytokines such as IL-6 and TNF-α. 

In the current study, the expression of inflammatory cytokines was higher in the OTTP (breast and thyroid) when compared to their healthy controls. Also, their levels of expression were associated with the over-expression of viral factors including *E2*, *E6*, and *E7*. These mentioned inflammatory cytokines (IL-1, IL-6, TNF- α, and TGF-β) not only are involved in inflammation but also are role player factors in the angiogenesis (Keshavarz et al., 2010; Norooznezhad et al., 2014). It has been shown that tumor angiogenesis is correlated with the invasiveness and metastasis in patients with breast cancer (Weidner et al., 1991).

The obtained results from the current study using advanced predictive regression, SEM, confirmed our previous data, which evaluated the direct and indirect effects of viral proteins and their interactions with cellular factors on the tumor development. However, structural equation modeling has some limitations such as dealing with missing data and small sample size in this study.

EBV infection and the induction of inflammatory factors have previously been shown to be associated with breast cancer (Richardson et al., 2015). In this study, the presence of EBV and HPV in breast cancer significantly increased expression of the pro-carcinogenic factors, TGF-β and IL-6 (and IL-6-related IL-11) in breast tissue compared with viral-negative tissue. Furthermore, levels of the pro-inflammatory factors IL-1 and TNF-α, the pro-proliferative transcription factor, NF-κB, as well as ROS and RNS levels, were significantly increased in viral positive compared with viral negative breast tissue Previously, other studies investigated the effect of EBV load in the sera and tumor tissue on the survival of patients with breast cancer (Marrão et al., 2014). The expression of *P53, RB, BRCA1*, and *BRCA2* were decreased in HPV-positive patients with breast cancer compared to the same patients with HPV-negative results and healthy controls. Also, it was shown that the presence of the HPV was associated with increased inflammatory cytokines (IL-1, IL-6, IL-17, TGF-β, TNF-α, and NF-kB) and tumor progression.

In conclusion, taken to gather, this study demonstrated that the presence of EBV and HPV in tumoral tissue could significantly increase the expression of a number of anoikis resistance, cellular signaling, and inflammatory genes which are important factors in the development of breast and thyroid cancers.

## References

[B1] Al Moustafa A-E, Chen D, Ghabreau L (2009). Association between human papillomavirus and Epstein-Barr virus infections in human oral carcinogenesis. Med Hypotheses.

[B2] Anastasiadi Z, Lianos GD, Ignatiadou E (2017). Breast cancer in young women: an overview. Updates Surg.

[B3] Bell AI, Groves K, Kelly GL (2006). Analysis of Epstein–Barr virus latent gene expression in endemic Burkitt’s lymphoma and nasopharyngeal carcinoma tumour cells by using quantitative real-time PCR assays. J Gen Virol.

[B4] Chen AY, Levy L, Goepfert H (2001). The development of breast carcinoma in women with thyroid carcinoma. Cancer.

[B5] Esquivel-Velázquez M, Ostoa-Saloma P, Palacios-Arreola MI (2015). The role of cytokines in breast cancer development and progression. J Interferon Cytokine Res.

[B6] Etemadi A, Mostafaei S, Yari K (2017). Detection and a possible link between parvovirus B19 and thyroid cancer. Tumor Biol.

[B7] Ferlay J, Shin HR, Bray F (2010). Estimates of worldwide burden of cancer in 2008: GLOBOCAN 2008. Int J Cancer.

[B8] Fernandes JV, Fernandes TAAdM, De Azevedo JCV (2015). Link between chronic inflammation and human papillomavirus-induced carcinogenesis. Oncol Lett.

[B9] Giani C, Fierabracci P, Bonacci R (1996). Relationship between breast cancer and thyroid disease: relevance of autoimmune thyroid disorders in breast malignancy. J Clin Endocrinol Metabol.

[B10] Goldszmid RS, Dzutsev A, Trinchieri G (2014). Host immune response to infection and cancer: unexpected commonalities. Cell Host Microbe.

[B11] Haklar G, Sayin-Özveri E, Yüksel M (2001). Different kinds of reactive oxygen and nitrogen species were detected in colon and breast tumors. Cancer Lett.

[B12] Jackson S, Harwood C, Thomas M (2000). Role of Bak in UV-induced apoptosis in skin cancer and abrogation by HPV E6 proteins. Genes Dev.

[B13] Jang M, Rhee J, Jang D-H (2011). Gene expression profiles are altered in human papillomavirus-16 E6 D25E-expressing cell lines. Virol J.

[B14] Kan C, Iacopetta B, Lawson J (2005). Identification of human papillomavirus DNA gene sequences in human breast cancer. Br J Cancer.

[B15] Keshavarz M, Norooznezhad AH, Mansouri K (2010). Cannabinoid (JWH-133) therapy could be effective for treatment of corneal neovascularization. Iran J Med Hypotheses Ideas.

[B16] Khodabandehlou N, Mostafaei S, Etemadi A (2019). Human papilloma virus and breast cancer: the role of inflammation and viral expressed proteins. BMC Cancer.

[B18] Kubota N, Wada K, Ito Y (2008). One-step multiplex real-time PCR assay to analyse the latency patterns of Epstein-Barr virus infection. J Virol Method.

[B19] Lu C, Heldt JM, Guille-Collignon M (2014). Quantitative analyses of ROS and RNS production in breast cancer cell lines incubated with ferrocifens. Chem Med Chem.

[B20] Marrão G, Habib M, Paiva A (2014). Epstein-Barr virus infection and clinical outcome in breast cancer patients correlate with immune cell TNF-α/IFN-γ response. BMC Cancer.

[B21] Moghoofei M, Mostafaei S, Nesaei A (2019). Epstein¨CBarr virus and thyroid cancer: The role of viral expressed proteins. J Cell Physiol.

[B22] Morris MA, Dawson CW, Wei W (2008). Epstein–Barr virus-encoded LMP1 induces a hyperproliferative and inflammatory gene expression programme in cultured keratinocytes. J Gen Virol.

[B23] Morshed K, Polz-Gruszka D, Szymański M (2014). Human papillomavirus (HPV)–structure, epidemiology and pathogenesis. Otolaryngol Pol.

[B24] Nicolini A, Carpi A, Rossi G (2006). Cytokines in breast cancer. Cytokine Growth Factor Rev.

[B25] Norooznezhad AH, Norooznezhad F, Ahmadi K (2014). Next target of tranilast: inhibition of corneal neovascularization. Med Hypotheses.

[B26] Pang M-F, Lin K-W, Peh S-C (2009). The signaling pathways of Epstein-Barr virus-encoded latent membrane protein 2A (LMP2A) in latency and cancer. Cell Mol Biol Lett.

[B27] Parkin DM (2006). The global health burden of infection-associated cancers in the year 2002. Int J Cancer.

[B28] Port RJP (2013). Epstein¨CBarr virus induction of the Hedgehog signalling pathway imposes a stem cell phenotype on human epithelial cells. J Pathol.

[B29] Rajarajan A, Stokes A, Bloor BK (2012). CD44 expression in oro-pharyngeal carcinoma tissues and cell lines. PLoS One.

[B30] Richardson AK, Currie MJ, Robinson BA (2015). Cytomegalovirus and Epstein-Barr virus in breast cancer. PLoS One.

[B31] Shannon-Lowe C, Adland E, Bell AI (2009). Features distinguishing Epstein-Barr virus infections of epithelial cells and B cells: viral genome expression, genome maintenance, and genome amplification. J Virol.

[B32] Shimakage M, Kawahara K, Sasagawa T (2003). Expression of Epstein-Barr virus in thyroid carcinoma correlates with tumor progression. Hum Pathol.

[B33] Siegel RL, Miller KD, Jemal A (2019). Cancer statistics, 2019. CA Cancer J Clin.

[B34] Sobel ME (1987). Direct and indirect effects in linear structural equation models. Soc Method Res.

[B35] Stamatiou DP, Derdas SP, Zoras OL (2016). Herpes and polyoma family viruses in thyroid cancer. Oncol Lett.

[B36] Stone SC, Rossetti RAM, Lima AM (2014). HPV associated tumor cells control tumor microenvironment and leukocytosis in experimental models. Immun Inflamm Dis.

[B37] Tomarken AJ, Waller NG (2005). Structural equation modeling: Strengths, limitations, and misconceptions. Annu Rev Clin Psychol.

[B38] van Baarle D, Hovenkamp E, Kersten MJ (1999). Direct Epstein-Barr virus (EBV) typing on peripheral blood mononuclear cells: no association between EBV type 2 infection or superinfection and the development of acquired immunodeficiency syndrome–related non-Hodgkin’s lymphoma. Blood.

[B39] Wang-Johanning F, Lu DW, Wang Y (2002). Quantitation of human papillomavirus 16 E6 and E7 DNA and RNA in residual material from ThinPrep Papanicolaou tests using real-time polymerase chain reaction analysis. Cancer.

[B40] Webster K, Parish J, Pandya M (2000). The human papillomavirus (HPV) 16 E2 protein induces apoptosis in the absence of other HPV proteins and via a p53-dependent pathway. J Biol Chem.

[B41] Weidner N, Semple JP, Welch WR (1991). Tumor angiogenesis and metastasis-correlation in invasive breast carcinoma. New Eng J Med.

[B42] Wolfle LM (1980). Strategies of path analysis. Am Edu Res J.

[B43] Wong M, Pagano JS, Schiller JT (2002). New associations of human papillomavirus, Simian virus 40, and Epstein-Barr virus with human cancer. J Natl Cancer Inst.

[B44] Yim E-K, Park J-S (2005). The role of HPV E6 and E7 oncoproteins in HPV-associated cervical carcinogenesis. Cancer Res Treat.

[B45] Zhang Y, Fan S, Meng Q (2005). BRCA1 interaction with human papillomavirus oncoproteins. J Biol Chem.

[B46] Zur Hausen H (2002). Papillomaviruses and cancer: from basic studies to clinical application. Nat Rev Cancer.

